# Supplementary material for: Differential and Overlapping Effects of Melatonin and Its Metabolites on Keratinocyte Function: Bioinformatics and Metabolic Analyses
Source: Antioxidants (Basel). 2021 Apr 17;10(4):618. doi: 10.3390/antiox10040618 (PMC8073250; doi:10.3390/antiox10040618)

Supplementary Material

- **Supplementary Tables**

Supplementary Table 1. Summary of Ingenuity Pathway Analysis (IPA) for top biological functions regulated by melatonin and its metabolites based on the data obtained from primary human epidermal keratinocytes. (0 – lack of activation, Z – score in IPA)

Compound	Categories	Diseases or Functions Annotation	*P*-value	Activation Z-score	# Molecules
**Melatonin**	Cancer, Dermatological Diseases and Conditions, Organismal Injury and Abnormalities	Cutaneous melanoma	4.3×10^{-21}	0	1016
	Cancer, Dermatological Diseases and Conditions, Organismal Injury and Abnormalities	Skin tumor	4.64×10^{-18}	1.29	1052
	Dermatological Diseases and Conditions, Organismal Injury and Abnormalities	Skin lesion	5.6×10^{-18}	-0.14	1058
	Cancer, Dermatological Diseases and Conditions, Organismal Injury and Abnormalities	Skin cancer	5.93×10^{-18}	0.39	1046
	Organismal Injury and Abnormalities, Reproductive System Disease	Nonobstructive azoospermia	3.49×10^{-13}	0	21
	Endocrine System Disorders, Gastrointestinal Disease, Metabolic Disease, Organismal Injury and Abnormalities	Diabetes mellitus	9.57×10^{-13}	-1.06	215
	Cancer, Dermatological Diseases and Conditions, Organismal Injury and Abnormalities	Skin squamous cell carcinoma	5.5×10^{-12}	0	159
	Cancer, Organismal Injury and Abnormalities	Melanoma	3.44×10^{-11}	0.79	1119
	Metabolic Disease, Organismal Injury and Abnormalities	Glucose metabolism disorder	2.09×10^{-10}	-0.67	247
	Cancer, Dermatological Diseases and Conditions, Organismal Injury and Abnormalities	Skin carcinoma	1.05×10^{-09}	0	229
	Cell-To-Cell Signaling and Interaction	Communication of cells	2.19×10^{-09}	-1.66	217
	Cardiovascular Disease, Organismal Injury and Abnormalities	Stenosis of artery	2.25×10^{-09}	0	31
	Organismal Injury and Abnormalities	Discomfort	3.23×10^{-09}	-0.01	73
	Cell-To-Cell Signaling and Interaction	Signal transduction	4.42×10^{-09}	0	199
	Organismal Injury and Abnormalities	Chronic pain	7.71×10^{-09}	0	31
	Respiratory Disease	Chronic respiratory disorder	1.11×10^{-08}	0.37	55
	Connective Tissue Disorders, Inflammatory Disease, Inflammatory Response, Organismal Injury and Abnormalities, Skeletal and Muscular Disorders	Osteoarthritis	1.15×10^{-08}	0	59
	Psychological Disorders	Drug dependence	2.11×10^{-08}	0	36
	Organismal Injury and Abnormalities	Non-cancer pain	2.91×10^{-08}	0	38
	Psychological Disorders	Alcoholism	3.15×10^{-08}	0	32
	Cardiovascular Disease, Organismal Injury and Abnormalities	Vaso-occlusion	3.78×10^{-08}	0.50	112
	Cardiovascular Disease	Occlusion of blood vessel	4.49×10^{-08}	0.46	110

	Gene Expression	Gene silencing	5.8×10^{-08}	0	25
	Gastrointestinal Disease	Nausea	6.37×10^{-08}	0	26
	Cardiovascular Disease, Organismal Injury and Abnormalities	Stenosis of aorta	7.15×10^{-08}	0	24
	Cancer, Organismal Injury and Abnormalities, Reproductive System Disease	Early stage invasive cervical squamous cell carcinoma	8.41×10^{-08}	0	11
	Cardiovascular Disease, Organismal Injury and Abnormalities	Infarction of heart	1.16×10^{-07}	-2.23	56
	Cardiovascular Disease, Organismal Injury and Abnormalities	Occlusion of artery	1.21×10^{-07}	0.10	107
	Neurological Disease, Psychological Disorders	Mania	1.7×10^{-07}	0	18
	Developmental Disorder, Hereditary Disorder, Ophthalmic Disease, Organismal Injury and Abnormalities	Recessive congenital stationary night blindness	1.94×10^{-07}	0	8
	Cell-To-Cell Signaling and Interaction, Nervous System Development and Function	Action potential of cells	2.08×10^{-07}	0.60	39
	Organismal Injury and Abnormalities	Backache	2.37×10^{-07}	0	23
	Cardiovascular Disease, Neurological Disease, Organismal Injury and Abnormalities, Psychological Disorders	Vascular dementia	2.78×10^{-07}	0	17
	Cardiovascular Disease, Organismal Injury and Abnormalities, Skeletal and Muscular Disorders	Myocardial infarction	2.9×10^{-07}	-1.80	54
	Cardiovascular Disease, Organismal Injury and Abnormalities	Acute coronary syndrome	3.2×10^{-07}	0	40
	Gastrointestinal Disease	Vomiting	3.63×10^{-07}	0	27
	Cardiovascular Disease, Organismal Injury and Abnormalities	Ischemia of heart	3.71×10^{-07}	0	46
	Developmental Disorder, Hereditary Disorder, Ophthalmic Disease, Organismal Injury and Abnormalities	Congenital stationary night blindness	4.27×10^{-07}	0	9
	Neurological Disease, Organismal Injury and Abnormalities	Subarachnoid hemorrhage	4.35×10^{-07}	0	20
	Cell-To-Cell Signaling and Interaction	Activation of cells	4.73×10^{-07}	0.04	183
	Cardiovascular Disease, Organismal Injury and Abnormalities	Infarction	5.89×10^{-07}	-1.96	80
	Cardiovascular Disease, Organismal Injury and Abnormalities	Disorder of coronary artery	6.36×10^{-07}	1.97	65
	Cell Signaling, Molecular Transport, Vitamin and Mineral Metabolism	Mobilization of Ca^{2+}	7.55×10^{-07}	0.66	66
	Inflammatory Response, Respiratory Disease	Inflammation of respiratory system component	8.61×10^{-07}	-0.30	118
**6(OH)Mel**	Cancer, Dermatological Diseases and Conditions, Organismal Injury and Abnormalities	Cutaneous melanoma	8.62×10^{-21}	0	1012
	Cancer, Dermatological Diseases and Conditions, Organismal Injury and Abnormalities	Skin cancer	1.07×10^{-17}	1.17	1042
	Dermatological Diseases and Conditions, Organismal Injury and Abnormalities	Skin lesion	2.37×10^{-17}	-0.53	1052
	Cancer, Dermatological Diseases and Conditions, Organismal Injury and Abnormalities	Skin tumor	3.04×10^{-17}	0.84	1045

	Cancer, Dermatological Diseases and Conditions, Organismal Injury and Abnormalities	Skin squamous cell carcinoma	1.46×10^{-14}	0	167
	Cancer, Dermatological Diseases and Conditions, Organismal Injury and Abnormalities	Non-melanoma skin cancer	2.15×10^{-14}	0	169
	Cancer, Organismal Injury and Abnormalities	Melanoma	1.49×10^{-12}	0.28	1125
	Cancer, Dermatological Diseases and Conditions, Organismal Injury and Abnormalities	Skin carcinoma	3.69×10^{-11}	0	235
	Molecular Transport	Quantity of metal	3.08×10^{-10}	1.88	109
	Molecular Transport	Quantity of metal ion	3.11×10^{-10}	1.95	98
	Organismal Injury and Abnormalities	Discomfort	4.77×10^{-10}	-0.41	75
	Cell-To-Cell Signaling and Interaction	Communication of cells	1.71×10^{-09}	-0.46	217
	Organismal Injury and Abnormalities, Reproductive System Disease	Nonobstructive azoospermia	6.37×10^{-09}	0	17
	Cell-To-Cell Signaling and Interaction	Signal transduction	9.67×10^{-09}	0	197
	Cell Signaling, Molecular Transport, Vitamin and Mineral Metabolism	Quantity of Ca^{2+}	1.23×10^{-08}	1.25	85
	Molecular Transport	Transport of ion	$1.87\times x10^{-08}$	0.66	113
	Molecular Transport	Transport of cation	2.58×10^{-08}	0.69	86
	Cell-To-Cell Signaling and Interaction, Nervous System Development and Function	Neurotransmissi on	2.67×10^{-08}	0.81	93
	Organismal Injury and Abnormalities, Psychological Disorders	Anxiety Disorders	4.25×10^{-08}	0	35
	Endocrine System Disorders, Gastrointestinal Disease, Metabolic Disease, Organismal Injury and Abnormalities	Diabetes mellitus	6.1×10^{-08}	-1.64	194
	Psychological Disorders	Alcoholism	1.09×10^{-07}	0	31
	Endocrine System Disorders, Gastrointestinal Disease, Immunological Disease, Metabolic Disease, Organismal Injury and Abnormalities	Insulin-dependent diabetes mellitus	1.6×10^{-07}	-1.89	86
	Cardiovascular Disease, Neurological Disease, Organismal Injury and Abnormalities	Stroke	2.03×10^{-07}	1.09	58
	Psychological Disorders	Drug dependence	2.23×10^{-07}	0	34
	Cell Morphology, Cellular Function and Maintenance	Depolarization of cells	2.51×10^{-07}	-2.63	18
	Nucleic Acid Metabolism, Small Molecule Biochemistry	Biosynthesis of cyclic nucleotides	2.91×10^{-07}	0.92	46
	Cardiovascular Disease, Organismal Injury and Abnormalities	Stenosis of aorta	3.1×10^{-07}	0	23
	Cellular Function and Maintenance	Ion homeostasis of cells	3.82×10^{-07}	-0.51	116
	Cardiovascular Disease, Organismal Injury and Abnormalities	Stenosis of artery	5.74×10^{-07}	0	27
	Behavior	Emotional behavior	6.13×10^{-07}	0.92	64
	Organismal Injury and Abnormalities	Non-cancer pain	8.32×10^{-07}	0	35
	Molecular Transport	Transport of inorganic cation	1.03×10^{-06}	0.02	74

	Respiratory Disease	Sleep disordered breathing	1.76×10^{-06}	0	24
	Metabolic Disease, Organismal Injury and Abnormalities	Glucose metabolism disorder	2.57×10^{-06}	-1.97	225
	Organismal Injury and Abnormalities	Progressive fibrosis	2.67×10^{-06}	0	10
	Connective Tissue Disorders, Organismal Injury and Abnormalities, Respiratory Disease	Progressive interstitial lung disease	2.67×10^{-06}	0	10
	Cellular Function and Maintenance, Molecular Transport	Flux of ion	2.75×10^{-06}	-0.41	68
	Connective Tissue Disorders, Inflammatory Disease, Inflammatory Response, Organismal Injury and Abnormalities, Respiratory Disease	Slowly progressive idiopathic pulmonary fibrosis	2.83×10^{-06}	0	8
	Cardiovascular Disease, Organismal Injury and Abnormalities	Aortic occlusive disease	3.35×10^{-06}	-1.38	30
	Cell Signaling, Nucleic Acid Metabolism, Small Molecule Biochemistry	Synthesis of cyclic AMP	3.69×10^{-06}	1.64	40
	Cellular Function and Maintenance, Molecular Transport	Flux of cation	3.83×10^{-06}	-0.79	65
	Cell-To-Cell Signaling and Interaction, Nervous System Development and Function	Action potential of cells	4.32×10^{-06}	0.11	36
	Gastrointestinal Disease	Vomiting	4.44×10^{-06}	0	25
	Cellular Function and Maintenance, Molecular Transport	Flux of inorganic cation	4.59×10^{-06}	-0.89	64
**AFMK**	Cancer, Dermatological Diseases and Conditions, Organismal Injury and Abnormalities	Cutaneous melanoma	2.06×10^{-23}	0	1059
	Dermatological Diseases and Conditions, Organismal Injury and Abnormalities	Skin lesion	8.72×10^{-22}	0.20	1111
	Cancer, Dermatological Diseases and Conditions, Organismal Injury and Abnormalities	Skin tumor	1.03×10^{-21}	0.46	1104
	Cancer, Dermatological Diseases and Conditions, Organismal Injury and Abnormalities	Skin cancer	1.24×10^{-21}	-0.15	1098
	Cancer, Dermatological Diseases and Conditions, Organismal Injury and Abnormalities	Skin squamous cell carcinoma	5.19×10^{-16}	0	176
	Cancer, Organismal Injury and Abnormalities	Melanoma	9.45×10^{-16}	1.09	1184
	Cancer, Dermatological Diseases and Conditions, Organismal Injury and Abnormalities	Skin carcinoma	4.42×10^{-15}	0	258
	Endocrine System Disorders, Gastrointestinal Disease, Metabolic Disease, Organismal Injury and Abnormalities	Diabetes mellitus	1.56×10^{-12}	-1.24	220
	Metabolic Disease, Organismal Injury and Abnormalities	Glucose metabolism disorder	4.72×10^{-11}	-0.78	257
	Organismal Injury and Abnormalities, Reproductive System Disease	Nonobstructive azoospermia	1.13×10^{-10}	0	19
	Molecular Transport	Transport of ion	7.72×10^{-10}	-0.21	121
	Cellular Function and Maintenance	Ion homeostasis of cells	1.06×10^{-09}	-0.15	129
	Organismal Injury and Abnormalities	Discomfort	2.56×10^{-09}	-0.43	75
	Molecular Transport	Transport of cation	3.46×10^{-09}	-0.27	91

	Respiratory Disease	Chronic respiratory disorder	1.4×10^{-08}	1.35	56
	Organismal Injury and Abnormalities, Respiratory Disease	Chronic obstructive pulmonary disease	2.06×10^{-08}	1.35	48
	Molecular Transport	Transport of K^+	5.21×10^{-08}	-0.15	30
	Molecular Transport	Transport of inorganic cation	1.26×10^{-07}	-0.59	79
	Cellular Function and Maintenance, Molecular Transport	Flux of ion	1.38×10^{-07}	0.007	74
	Cellular Function and Maintenance, Molecular Transport	Flux of inorganic cation	2.04×10^{-07}	-0.53	70
	Cell Signaling, Molecular Transport, Vitamin and Mineral Metabolism	Mobilization of Ca^{2+}	2.71×10^{-07}	-0.27	69
	Molecular Transport	Transport of monovalent inorganic cation	4.09×10^{-07}	-0.15	49
	Nervous System Development and Function	Olfactory response	4.41×10^{-07}	0	18
	Cellular Movement, Hematological System Development and Function, Immune Cell Trafficking, Inflammatory Response	Chemotaxis of antigen presenting cells	4.56×10^{-07}	0.28	40
	Inflammatory Response, Respiratory Disease	Inflammation of respiratory system component	5.71×10^{-07}	-0.35	122
	Molecular Transport	Transport of metal ion	6.42×10^{-07}	-0.37	71
	Nervous System Development and Function	Olfaction	6.58×10^{-07}	-0.43	24
	Molecular Transport	Quantity of metal	9.31×10^{-07}	-0.54	100
	Molecular Transport	Quantity of metal ion	1.16×10^{-06}	-0.34	89
	Cellular Movement, Hematological System Development and Function, Immune Cell Trafficking, Inflammatory Response	Chemotaxis of phagocytes	1.43×10^{-06}	-0.14	66
	Inflammatory Response, Organismal Injury and Abnormalities	Inflammation of organ	1.56×10^{-06}	-0.04	247
	Cancer, Organismal Injury and Abnormalities, Reproductive System Disease	Early stage invasive cervical squamous cell carcinoma	1.73×10^{-06}	0	10
	Inflammatory Response	Inflammation of body cavity	1.92×10^{-06}	-0.25	186
	Gene Expression	Gene silencing	1.96×10^{-06}	0	23
	Cell Signaling, Cellular Function and Maintenance, Molecular Transport, Vitamin and Mineral Metabolism	Flux of Ca^{2+}	1.97×10^{-06}	-0.27	64
	Cardiovascular Disease, Organismal Injury and Abnormalities	Ischemia of heart	2.45×10^{-06}	0	45
	Connective Tissue Disorders, Organismal Injury and Abnormalities, Respiratory Disease	Progressive interstitial lung disease	3.76×10^{-06}	0	10

	Connective Tissue Disorders, Inflammatory Disease, Inflammatory Response, Organismal Injury and Abnormalities, Respiratory Disease	Slowly progressive idiopathic pulmonary fibrosis	3.76×10^{-06}	0	8
	Organismal Injury and Abnormalities	Stenosis of vasculature	3.92×10^{-06}	0.93	27
	Inflammatory Response	Inflammation of absolute anatomical region	3.94×10^{-06}	-0.66	211
	Cardiovascular Disease, Organismal Injury and Abnormalities	Vaso-occlusion	3.96×10^{-06}	0.26	107
	Cell-To-Cell Signaling and Interaction	Communication of cells	4.11×10^{-06}	-0.65	206
	Cardiovascular Disease, Organismal Injury and Abnormalities	Stenosis of artery	4.12×10^{-06}	1.40	26
	Inflammatory Disease, Inflammatory Response, Organismal Injury and Abnormalities, Respiratory Disease	Inflammation of lung	4.28×10^{-06}	-0.43	86

- **Supplementary Figures**

Supplementary Figure 1. Volcano Plot diagram of differentially expressed genes for melatonin *vs* EtOH (**A**), 6-hydroxymelatonin *vs* EtOH (**B**), and AFMK *vs* EtOH (**C**).

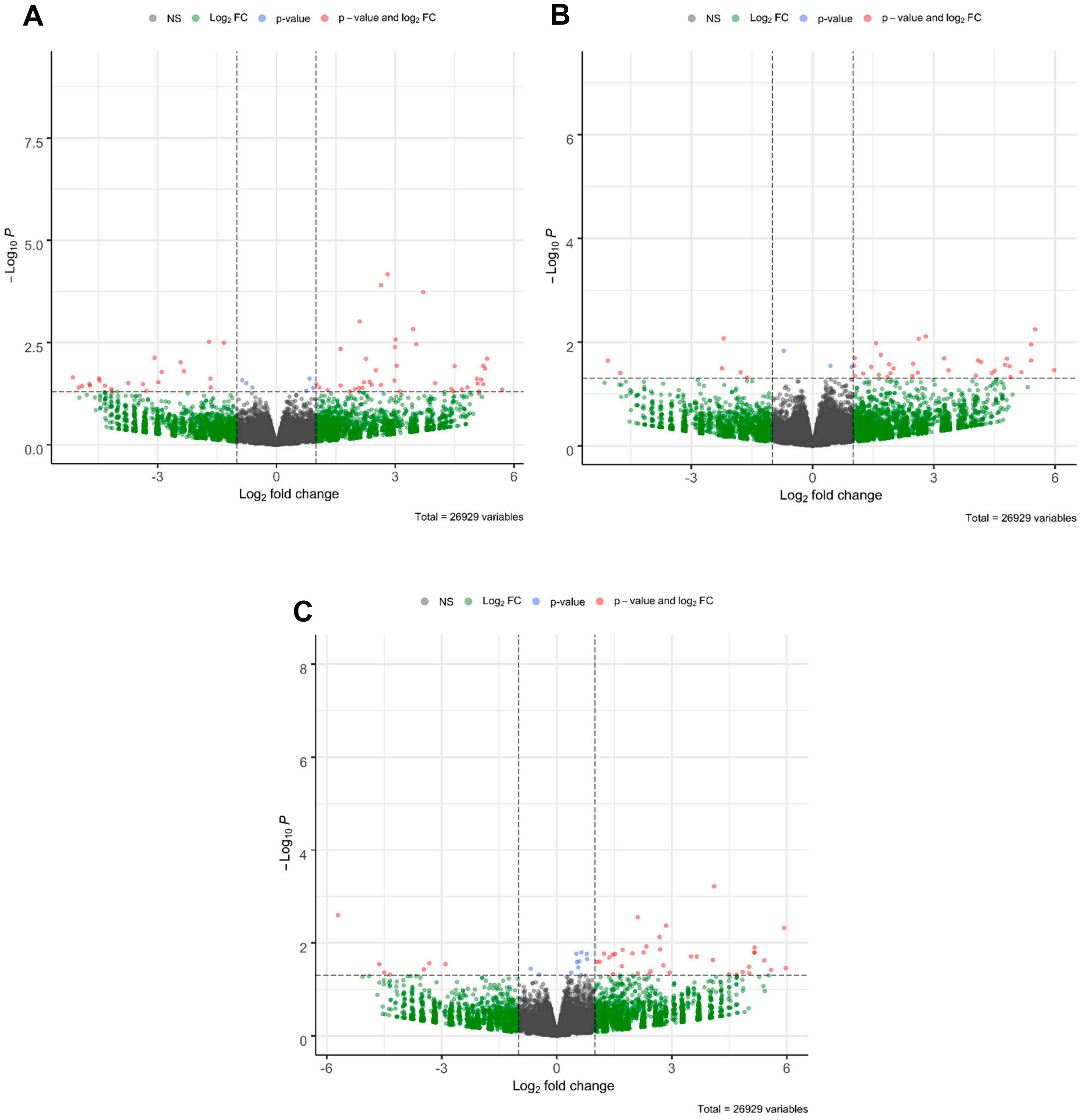

Supplement: Supplementary file 1 [file antioxidants-10-00618-s001.zip › antioxidants-1133847-supplementary.pdf]
